# Construction and characterization of stable, constitutively expressed, chromosomal green and red fluorescent transcriptional fusions in the select agents, *Bacillus anthracis, Yersinia pestis, Burkholderia mallei*, and *Burkholderia pseudomallei*

**DOI:** 10.1002/mbo3.192

**Published:** 2014-07-09

**Authors:** Shengchang Su, Hansraj Bangar, Roland Saldanha, Adin Pemberton, Bruce Aronow, Gary E Dean, Thomas J Lamkin, Daniel J Hassett

**Affiliations:** 1 Department of Molecular Genetics, Biochemistry and Microbiology, University of Cincinnati College of MedicineCincinnati, Ohio, 45267; 2 UES, Inc.Dayton, Ohio, 45432; 3 Division of Biomedical Informatics, Cincinnati Children's Hospital Medical CenterCincinnati, Ohio, 45229-3039; 4 Air Force Research Laboratory, 711th HPW/RHXBC, Molecular Signatures SectionWright-Patterson AFB, Ohio, 45433-7913

**Keywords:** GFP, RFP, fluorescent tagging, select agents, *Bacillus anthracis*., *Yersinia pestis*, *Burkholderia mallei*, *Burkholderia pseudomallei*

## Abstract

Here, we constructed stable, chromosomal, constitutively expressed, green and red fluorescent protein (GFP and RFP) as reporters in the select agents, *Bacillus anthracis, Yersinia pestis, Burkholderia mallei*, and *Burkholderia pseudomallei*. Using bioinformatic approaches and other experimental analyses, we identified P0253 and P1 as potent promoters that drive the optimal expression of fluorescent reporters in single copy in *B. anthracis* and *Burkholderia* spp. as well as their surrogate strains, respectively. In comparison, *Y. pestis* and its surrogate strain need two chromosomal copies of *cysZK* promoter (P2*cysZK*) for optimal fluorescence. The P0253-, P2*cysZK-*, and P1-driven GFP and RFP fusions were first cloned into the vectors pRP1028, pUC18R6KT-mini-Tn*7*T-Km, pmini-Tn*7*-*gat*, or their derivatives. The resultant constructs were delivered into the respective surrogates and subsequently into the select agent strains. The chromosomal GFP- and RFP-tagged strains exhibited bright fluorescence at an exposure time of less than 200 msec and displayed the same virulence traits as their wild-type parental strains. The utility of the tagged strains was proven by the macrophage infection assays and lactate dehydrogenase release analysis. Such strains will be extremely useful in high-throughput screens for novel compounds that could either kill these organisms, or interfere with critical virulence processes in these important bioweapon agents and during infection of alveolar macrophages.

## Introduction

The bacterial pathogens *Bacillus anthracis, Yersinia pestis, Burkholderia mallei*, and *Burkholderia pseudomallei* are the etiologic agents of the diseases anthrax, plague, glanders, and melioidosis, respectively. These organisms are listed by the Centers for Disease Control and Prevention (CDC) as high-priority biological agents that pose a risk to national security because they (i) can be easily disseminated or transmitted; (ii) result in high mortality rates and have the potential for major public health impact; (iii) may elicit public panic and social disruption; and (iv) require special action for public health preparedness. Based on how easily they can be spread and the severity of illness they cause, *B. anthracis, Y. pestis, B. mallei*, and *B. pseudomallei* are classified as Tier 1 select agents.

The horrific events of 11 September 2001 and the subsequent anthrax attacks that caused illness and several deaths have demonstrated that the world needs to be prepared for an increasing number of terrorist attacks, which may include the use of potential biological warfare agents. Many U.S. agencies such as APHIS, CDC, NIAID, DTRA, and HHS started an initiative intended to better understand the pathogenic mechanisms of these organisms and to promote public health and safety by providing effective vaccines and new treatment options. Among research tools to achieve these goals, high-throughput screening (HTS) is a valuable assay to discover compounds that have antimicrobial properties using commercially available chemical compound libraries. In addition, intramacrophage survival assays using genome-wide bacterial mutants are critical to unravel the mechanism of pathogenesis of the aforementioned organisms. However, both HTS analysis and large scale of intramacrophage survival measurements require a rapid, accurate, and reproducible reporting system.

Since the gene encoding green fluorescent protein (GFP) from the jellyfish (*Aequorea victoria*) was cloned and expressed in both prokaryotic (*Escherichia coli*) or eukaryotic (*Caenorhabditis elegans*) cells (Chalfie et al. 1994), fluorescent proteins have been powerful investigative tools in deciphering biological processes and thus have been widely used as marker systems for prokaryotic organisms (Parker and Bermudez 1997; Valdivia and Falkow 1997; Bumann 2001; Poschet et al. 2001). One of important applications of fluorescent proteins was bacterial tagging, which enables tracking of bacteria in complex environments, including cellular infection, especially by intracellular pathogens. To obtain the highest levels of fluorescence, most researchers have employed relatively facile and efficient means to utilize a replicative plasmid-borne fluorescent reporter to label and track the bacteria in vitro or in vivo (e.g., within macrophages). However, the stability and proper maintenance of the reporter plasmids in these bacteria and the antibiotic resistance conferred by the plasmids within the host can become highly problematic because some antibiotics that are approved for use in select agents such as *B. anthracis* Ames, *Y. pestis, B. mallei*, and *B. pseudomallei* research do not enter human cells. In addition, the restricted use of antibiotic markers in select agents and the inherent antibiotic resistance of these organisms add another level of complexity to the fluorescent tagging of these species. Hence, it is preferable to tag the bacterial cells with a marker gene that is stably integrated into the bacterial chromosome in order to reduce the risk of marker loss or marker transfer to other species. To date, chromosomally GFP- or RFP-tagged *Y. pestis, B. mallei*, and *B. pseudomallei* have been described (Choi et al. 2005; Norris et al. 2010; Bland et al. 2011). However, due to the weakness of promoter driving the expression of GFP and RFP in these respective organisms, they are not suitable for high-throughput analyses, which require less than ∼200 msec exposure time. Thus, the lack of fluorescent *B. anthracis, Y. pestis, B. mallei*, and *B. pseudomallei* appropriate for rapid, large scale, HTS analysis necessitates further improvement.

In this report, we demonstrate the generation of stable, constitutively expressed, chromosomal transcriptional GFP and RFP fusions in each of the four aforementioned strains that allow for evaluation of fluorescence using the statistical minimum of a 200 msec exposure time. Such strains will be extremely valuable reagents for researchers around the world to screen candidate compounds and/or chemical libraries for antibacterial activity in the event of a bioterrorist attack and/or a developing trend toward increased antibiotic resistance in virulent strains.

## Materials and Methods

### Bacterial strains and growth media

Bacterial strains used in this study are listed in Table1. All cloning was conducted in *E. coli* DH5*α*, DH5*α λ*pir, or EPmax10B (Bio-Rad, Hercules, CA, USA.). *Escherichia coli* was routinely grown at 37°C in Luria–Bertani broth (LB, Life Technologies, Grand Island, NY, USA.) or 1× M9 minimal medium plus 20 mmol/L glucose (MG medium). When necessary, diaminopimelate (DAP) was supplemented at a final concentration of 200 *μ*g/mL. All work with the type A virulent strains *B. anthracis* Ames, *Y. pestis* CO92, *Burkholderia pseudomallei* K96243, *B. mallei* NBL7, and their derivatives were performed in a biosafety level 3 (BSL-3) facility using standard BSL-3 practices, procedures, and containment equipment that was approved by the Institutional Biosafety Committees of the University of Cincinnati (UC). UC is registered with the USDA and the CDC and Prevention to work with these highly virulent pathogens. The surrogate strains of the aforementioned select agents were *B. anthracis* Sterne, *Yersina pseudotuberculosis* (ATCC 11960), and *B. thailandensis* (ATCC 700388), respectively. *Bacillus anthracis* sp. and their derivatives were grown aerobically at 37°C in brain heart infusion (BHI) medium (Difco, Franklin Lakes, NJ, USA.). *Yersina pseudotuberculosis, Y*. *pestis*, and their derivatives were cultured aerobically at 37°C in tryptic soy broth (TSB, Difco). *Burkholderia thailandensis, B. pseudomallei, B. mallei*, and their derivatives were grown at 37°C in LB or MG medium. Antibiotics and nonantibiotic antibacterial compounds were added at the following concentrations when required. For *E. coli* strains: kanamycin, 50 *μ*g/mL; ampicillin, 100 *μ*g/mL; erythromycin, 300 *μ*g/mL; and glyphosate (GS, primary active ingredient of the herbicide Roundup™ containing 50.2% glyphosate, purchased from local hardware store), 0.3%; for *B. anthracis*: erythromycin, 5 *μ*g/mL; tetracycline, 10 *μ*g/mL; kanamycin, 20 *μ*g/mL; and spectinomycin, 250 *μ*g/mL; for *Y. pseudotuberculosis* and *Y*. *pestis*: kanamycin, 20 *μ*g/mL; chloramphenicol, 35 *μ*g/mL; for *B. thailandensis*: GS, 0.04%; for *B. pseudomallei*: GS, 0.3%; for *B. mallei*: GS, 0.2%.

**Table 1:** Bacterial strains used in this study.

Strain	Description (relevant genotype or phenotype)	Source or reference
*Escherichia coli* strains		
DH5*α*	F^*−*^ Φ80d*lacZ*Δ*M15 endA1 recA1 hsdR17(r*_*K*_^*−*^ *m*_*K*_^*−*^*) supE44 thi-1 gyrA96* Δ*(lacZYA-argF)*U169	Invitrogen
DH5*α λpir*	*λpir* lysogen of DH5*α*	Laboratory strain
S17-1	Pro^−^ Res^−^ Mod^+^ *recA*; integrated RP4-Tet::Mu-Kan::Tn*7*, Mob^+^	Simon et al. (1983)
S17-1 *λpir*	*λpir* lysogen of S17-1	Laboratory strain
SS1827	Helper strain for conjugation	Stibitz and Carbonetti (1994)
GM2163	*dam dcm* strain, deficient in adenine and cytosine methylation	Fermentas
RHO3	Km^s^; SM10(*λpir*) Δ*asd*::*FRT* Δ*aphA*::*FRT*	Lopez et al. (2009)
EPMax10B-*lacI*^*q*^*/pir/leu*^*+*^ (E1889)	F^−^*λ*^−^ *mcrA* Δ(*mrr-hsdRMS-mcrBC*) Φ80d*lacZ*Δ*M15* Δ*laX74 deoR endA1 recA1galU galK rpsL nupG 9 lacI*^*q*^*-FRT8 pir-FRT4*	Norris et al. (2009)
EPMax10B-*pir116*Δ*asd/*Δ*trp*::Gm^r^/*mob-*Km^*+*^ (E1354)	Gm^r^ Km^r^ F^−^*λ*^−^ *mcrA* Δ(*mrr-hsdRMS-mcrBC*) Φ80d*lacZ*Δ*M15* Δ*laX74 deoR endA1 recA1 araD139* Δ(*ara leu)7697 galU galK rpsL nupG Tn-pir116-FRT2*Δ*asd*::*wFRT* Δ*trp*::Gm^r^-*FRT5 mob* [*recA*::*RP4-2 Tc*::*Mu-Km*^*r*^]	Norris et al. (2009)
EPMax10B-Δ*dapA*:: *lacI*^*q*^*-pir-*Gm^r^/*mob-*Km^*+*^*/leu*^*+*^ (E2072)	Gm^r^ Km^r^ F^−^*λ*^−^ *mcrA* Δ(*mrr-hsdRMS-mcrBC*) Φ80d*lacZ*Δ*M15* Δ*laX74 deoR endA1 recA1 araD139* Δ(*ara leu)7697 galU galK rpsL nupG* Δ*dapA*::*pir-lacI*^*q*^*-*Gm^r^-*FRT8 mob* [*recA*::*RP4-2 Tc*::*Mu-Km*^*r*^] *leu*^*+*^	Zarzycki-Siek et al. (2013)
*Bacillus anthracis* strains		
Sterne	Surrogate strain, pXO_1_^+^/pXO_2_^−^	B. E. I. Resources NR-1400
Ames	Virulent type A *B. anthracis* strain, pXO_1_^+^/pXO_2_^+^	B. E. I. Resources NR-411, Little and Knudson (1986)
Sterne::P*ntr*-*gfp*	Δ*bla1*::p*ntr-gfp*, chromosomal GFP-tagged Sterne	This study
Sterne::P*ntr*-*rfp*	Δ*bla1*::p*ntr-rfp*, chromosomal RFP-tagged Sterne	This study
Sterne::P0253-*gfp*	Δ*bla1*::p0253*-gfp*, chromosomal GFP-tagged Sterne	This study
Sterne::P0253-*rfp*	Δ*bla1*::p0253*-rfp*, chromosomal RFP-tagged Sterne	This study
Ames::P0253-*gfp*	Δ*bla1*::p0253*-gfp*, chromosomal GFP-tagged Ames	This study
Ames::P0253-*rfp*	Δ*bla1*::p0253*-rfp*, chromosomal RFP-tagged Ames	This study
*Burkholderia* strains		
* B. pseudomallei* K96243	Sequenced prototype virulent strain, clinical isolate	B. E. I. Resources NR-4073, Holden et al. (2004)
* B. mallei* NBL7	Virulent *B. mallei* strain China 7, derived from ATCC 23344	B. E. I. Resources NR-4071
* B. thailandensis*	Surrogate strain E264, ATCC 700388	ATCC
* B. thailandensis*::P1-*gfp*	P1 integron promoter-driven *gfp* reporter fusion inserted at the chromosomal *glmS1 att-*Tn*7* site of *B. thailandensis*	This study
* B. thailandensis*::P1-*rfp*	P1 integron promoter-driven *rfp* reporter fusion inserted at the chromosomal *glmS1 att-*Tn*7* site of *B. thailandensis*	This study
* B. mallei*::P1-*gfp*	P1 integron promoter-driven *gfp* reporter fusion inserted at the chromosomal *glmS1 att-*Tn*7* site of *B. mallei*	This study
* B. mallei*::P1-*rfp*	P1 integron promoter-driven *rfp* reporter fusion inserted at the chromosomal *glmS1 att-*Tn*7* site of *B. mallei*	This study
* B. pseudomallei*::P1-*gfp*	P1 integron promoter-driven *gfp* reporter fusion inserted at the chromosomal *glmS1 att-*Tn*7* site of *B. pseud mallei*	This study
* B. pseudomallei*::P1-*rfp*	P1 integron promoter-driven *rfp* reporter fusion inserted at the chromosomal *glmS1 att-*Tn*7* site of *B. pseudomallei*	This study
*Yersinia* strains		This study
*Y. pseudotuberculosis*	Wild-type strain, ATCC# 11960 (Pfeiffer)	ATCC
*Y. pestis* CO92	Biovar Orientalis, pMT1^+^, pCD1^+^, pPCP1^+^	B. E. I. Resources NR-641, (Parkhill et al. 2001)
*Y. pseudotuberculosis*::*cysZK-gfp*	*cysZK*-driven *gfp* reporter fusion integrated into the chromosomal *att-*Tn*7* site of *Y. pseudotuberculosis*	This study
*Y. pseudotuberculosis*::*cysZK-rfp*	*cysZK*-driven *rfp* reporter fusion integrated into the chromosomal *att-*Tn*7* site of *Y. pseudotuberculosis*	This study
*Y. pseudotuberculosis*::2*cysZK-gfp*	Two copies of *cysZK*-driven *gfp* reporter fusion integrated into the chromosomal *att-*Tn*7* site of *Y. pseudotuberculosis*	This study
*Y. pseudotuberculosis*::2*cysZK-rfp*	Two copies of *cysZK*-driven *rfp* reporter fusion integrated into the chromosomal *att-*Tn*7* site of *Y. pseudotuberculosis*	This study
*Y. pestis*::2*cysZK-gfp*	Two copies of *cysZK*-driven *gfp* reporter fusion integrated into the chromosomal *att-*Tn*7* site of *Y. pestis* CO92	This study
*Y. pestis*::2*cysZK-rfp*	Two copies of *cysZK*-driven *rfp* reporter fusion integrated into the chromosomal *att-*Tn*7* site of *Y. pestis* CO92	This study

### DNA manipulations

Plasmids and oligonucleotides used in this study are listed in Tables2 and 3, respectively. Genomic DNA isolation, PCR, restriction enzyme digestion, ligation, cloning, and DNA electrophoresis were performed according to standard techniques (Maniatis et al. 1982). All oligonucleotide primers were synthesized by integrated DNA technologies (IDT). PCR was performed using either Choice *Taq* Mastermix (Denville Scientific, Inc. South Plainfield, NJ, USA) or *Pfu* DNA polymerase (Stratagene, La Jolla, CA, USA). Plasmids were prepared using a QIAprep Spin miniprep kits (Qiagen, Valencia, CA, USA.) as recommended by the manufacturer. DNA fragments were purified using either a QIAquick PCR purification kit (Qiagen) or a QIAquick gel extraction kit (Qiagen). All cloned inserts were confirmed by automated DNA sequencing performed at the DNA Core Facility of the Cincinnati Children's Hospital Medical Center. Plasmids were introduced into *E. coli* by CaCl_2_-mediated transformation and into *Bacillus* sp., *Yersinia* sp., and *Burkholderia* sp. by electroporation or conjugation.

**Table 2:** Plasmids used in this study.

Plasmid	Description	Source or reference
pBluescript SK^+^	High-copy cloning vector; Ap^r^	Invitrogen
pBKJ258	Tm^S^ allelic-exchange vector; Em^r^	Lee et al. (2007)
pBKJ223	I-SceI expression vector; Tc^r^, Ap^r^	Janes and Stibitz (2006)
pRP1028	Tm^S^ allelic-exchange vector; Sp^r^	Dr. Scott Stibitz
pRP1028m	*turbo-rfp*-minus pRP1028; Sp^r^	This study
pUC18R6KT-mini-Tn*7*T-Km	Ap^r^; Km^r^ on mini-Tn*7*T; R6K replicon and *oriT*	Choi et al. (2005)
pTNS2	Ap^r^; R6K replicon; encodes the TnsABC+D specific transposition pathway	Choi et al. (2005)
pTNS3-*asd*_*Ec*_	Helper plasmid containing *asd*_*Ec*_ for Tn*7* site-specific transposition system	Kang et al. (2009)
pFLP2	Ap^r^/Cb^r^; Flp recombinase expression vector	Becher and Schweizer (2000)
pBKJΔ*bla1*	2 kb flanking sequences of *bla1* were cloned between *Not*I-*Sac*II sites of pBKJ258; Em^r^	This study
pBKJΔ*bla1*::p*ntr*-*gfp*	Promoter p*ntr*-driven Superfolder *gfp* cloned into pBKJΔ*bla1*; Em^r^	This study
pBKJΔ*bla1*::p0253-*gfp*	Promoter p0253-driven Superfolder *gfp* cloned into pBKJΔ*bla1*; Em^r^	This study
pBKJΔ*bla1*::p*ntr*-*rfp*	Promoter p*ntr*-driven TurboRed *rfp* cloned into pBKJΔ*bla1*; Em^r^	This study
pBKJΔ*bla1*::p0253-*rfp*	Promoter p0253-driven TurboRed *rfp* cloned into pBKJΔ*bla1*; Em^r^	This study
pRP1028Δ*bla1*::p0253-*gfp*	Promoter p0253-driven Superfolder GFP cloned into pRP1028; Sp^r^	This study
pRP1028m^−^Δ*bla1*::p0253-*rfp*	Promoter p0253-driven TurboRed RFP cloned into pRP1028*rfp*^−^; Sp^r^	This study
pUC18R6KT-P*cysZK*-*gfp*	Promoter *cysZK-*driven *gfp* reporter fusion cloned between *Sma*I and *Apa*I sites of pUC18R6KT-mini-Tn*7*T-Km	This study
pUC18R6KT-P*cysZK*-*rfp*	Promoter *cysZK-*driven *rfp* reporter fusion cloned between *Sma*I and *Apa*I sites of pUC18R6KT-mini-Tn*7*T-Km	This study
pUC18R6KT-2P*cysZK*-*gfp*	Second copy of *cysZK-*driven *gfp* reporter fusion cloned between *Apa*I and *Kpn*I sites of pUC18R6KT-P*cysZK*-*gfp*	This study
pUC18R6KT-2P*cysZK*-*rfp*	Second copy of *cysZK-*driven *rfp* reporter fusion cloned between *Apa*I and *Kpn*I sites of pUC18R6KT-P*cysZK*-*rfp*	This study
pmini-Tn*7*-*gat*	mini-Tn*7* integration vector based on *gat*	Norris et al. (2009)
pmini-Tn*7*-*gat-*P1-*gfp*	Integron promoter P1-driven *egfp* reporter fusion cloned between *Hind*III and *Eco*RI sites of pmini-Tn*7*-*gat*	This study
pmini-Tn*7*-*gat-*P1-*rfp*	Integron promoter P1-driven *rfp* reporter fusion cloned between *Hind*III and *Eco*RI sites of pmini-Tn*7*-*gat*	This study

Km^r^, kanamycin resistance; Ap^r^, ampicillin resistance; Em^r^, erythromycin resistance; Cm^r^, chloramphenicol resistance; Sp^r^, spectinomycin resistance.

**Table 3:** Oligonucleotides used in this study.

Oligonucleotide	Sequence (5′ to 3′)	Restriction site
U*bla1/Not5*′	AAGGAAAAAAGCGGCCGCATACATGTTCCAGAC	*Not*I
U*bla1/Sm3*′	TCCCCCGGGACTAGGCTTGTAATAC	*Sma*I
D*bla1/Sm5*′	TCCCCCGGGTATCGTTTGGCCACC	*Sma*I
D*bla1/Sac5*′	TCCCCGCGGACCTGTTAACGCTGC	*Sac*II
*gfp*/*Pst*5′	AACTGCAGATGCGTAAAGGAGAAGAATTA	*Pst*I
*gfp*/*Apa*I3′	GGAATTCGGGCCCTTACTATTTGTATAA	*Apa*I
*gfp/Sm3*′	TCCCCCGGGTTACTATTTGTATAATTC	*Sma*I
*egfp/Pst*5′	AACTGCAGTGATTAACTTTATAAGGAGGAAAAACATATGAGTAAAGGAGAAG	*Pst*I
*egfp/Eco*3′	CGGAATTCTTATTTGTATAGTTCATCC	*Eco*RI
Turbo *rfp*/*Pst*5′	AACTGCAGATGAGCGAACTAATAAAG	*Pst*I
Turbo *rfp*/*Eco*3′	GGAATTCGTCGACCCGGGCTATTAACGGTGCCCTAATTTG	*Pst*I
Turbo *rfp/Sm3*′	TCCCCCGGGCTATTAACGGTGCCCTAATTTG	*Sma*I
Burk *rfp/Pst5*′	AACTGCAGTGATTAACTTTATAAGGAGGAAAAACATATGAGCGAGCTGATC	*Pst*I
Burk *rfp/Eco3*′	CGGAATTCTCACCGGTGCCCCAGCTTG	*Eco*RI
P*ntr*/*Sma*5′	TCCCCCGGGGATCTGATCA CTGAGTTGGA	*Sma*I
P*ntr*/*Pst*3′	AACTGCAGCATCATAATTCCCTCCAATTG	*Pst*I
P0253/*Sma*5′	TCCCCCGGGAAGGTAGTATGATTTGC	*Sma*I
P0253/*Pst*3′	AACTGCAGCAAAAATACACCTCCACCGTC	*Pst*I
*cysZK/Sm5*′	TAACCCGGGAATAAAGTCGATAACTTGCAATTCGG	*Sma*I
*cysZK/Pst3*′	AACTGCAGAACTCTATGAAAATGTAGGGAACG	*Pst*I
*cysZK/Apa5*′	TCCGGGCCCAATAAAGTCGATAACTTGC	*Apa*I
P1/*Hind*5′	CCCAAGCTTACTAGTGAACACGAAC	*Hind*III
P1/*Pst*3′	AACTGCAGTCGAATCCTTCTTGTGAATC	*Pst*I
YPatt5′	5′-GCCACATGTCGAAGAAATTATTGC	
YPatt3′	5′-TTGTAAAAAATTCAGCGTATCAG	
PTn*7*L	5′-ATTAGCTTACGACGCTACACCC	
PTn*7*R	5′-CACAGCATAACTGGACTGATTTC	
Pla5′	5′-ATAACTATTCTGTCCGGGAGTGC	
Pla3′	5′-TCAGAAGCGATATTGCAGACCC	
Ymt5′	5′-ATGACTGAAGTACTGCGGAATTCGC	
Ymt3′	5′-CCAAGCACTCACGAGATCTTGCTGTG	
LcrV5′	5′-GACGTGTCATCTAGCAGACG	
LcrV3′	5′-ATGATTAGAGCCTACGAACAAAACCC	
BTglmS1	5′-GTTCGTCGTCCACTGGGATCA	
BTglmS2	5′-AGATCGGATGGAATTCGTGGAG	
BPglmS1	5′-GAGGAGTGGGCGTCGATCAAC	
BPglmS2	5′-ACACGACGCAAGAGCGGAATC	
BPglmS3	5′-CGGACAGGTTCGCGCCATGC	
BMglmS1	5′-ACACGACGCAAAAGCGGAATC	
BMglmS2	5′-AGTGGGCGTCGATCAACGCG	

### Bioinformatic analyses

Several gene expression datasets (Liu et al. 2004; Rodrigues et al. 2006; Sebbane et al. 2006; Bergman et al. 2007; Vadyvaloo et al. 2010) (NCBI GEO database and/or ArrayExpress database) were used, respectively, to identify three groups of *B. anthracis, Y. pestis*, and *B. pseudomallei* gene transcripts that exhibited on average throughout the time series, high, medium, and moderately low expression based on RMA (Robust Multi-array Analysis)-normalized Affymetrix probe set intensity levels. Each of these groups was then ranked for those transcripts that had the least variance as a function of time during macrophage infection. Of the transcripts that were identified in each tier, their relative position was used on the genome to identify those that were most likely in the 5′ most position of a potential operon, and the sequence upstream of that was used to test for potential promoter activity that could drive high-, medium-, or low-level GFP expression, respectively (refer to Tables S1–S3).

### Macrophage preparation and bacterial infection

The human monocytic cell line, THP-1, was generously provided from William Miller (University of Cincinnati College of Medicine, Department of Molecular Genetics, Biochemistry and Microbiology). THP-1 cells were maintained in Roswell Park Memorial Institute (RPMI)-1640 medium supplemented with 10% v/v fetal bovine serum at 37°C in 5% CO_2_. Freshly propagated cells were seeded in 384 well plates at a density of 20,000 cells per well and incubated at 37°C to differentiate into macrophages following exposure to 80 nmol/L phorbol 12-myristate 13-acetate (PMA) for 3 days. Differentiated THP-1 cells were infected with 50 MOI of GFP-tagged *B. mallei, B. pseudomallei, Y. pestis*, and *B. anthracis* and phagocytized for 90 min. After infection, cells were washed thrice and treated with RPMI containing gentamicin (50 *μ*g/mL) for *Y. pestis* and *B. anthracis* or kanamycin (250 *μ*g/mL) for *B. mallei, B. pseudomallei* to kill extracellular bacteria. Cells were processed for various analyses including microscopic imaging, bacterial load determination (as CFU), and lactate dehydrogenase (LDH) assays at different time points (0, 12, 24, 48, and 72 h).

### Infection kinetics

At each time point, cells were harvested and lysed with 0.1% SDS. Differential bacterial load was determined by enumeration of colony forming units (CFU) at different time points.

### Cytotoxicity assays

Cytotoxicity was measured based on release of LDH by following CytoTox-ONE homogenous membrane integrity assay kit instruction manual (Promega, Madison, WI). Supernatant from different time points was used to measure cytotoxicity. Fluorescence was measured using an excitation wavelength of 560 nm and an emission wavelength of 590 nm following 10 sec of shaking. The results are presented as relative fluorescence units (RFU), which was then calculated as percentage of dead cells as compared to lysed cells.

### Image analysis

At each time point, cells were washed with phosphate-buffered saline (PBS) and fixed with 4% *para*-formaldehyde (PFA) at room temperature for 30 min. Cell images were captured at different magnifications using GFP (green 485/524 nm excitation/emission) filter and phase contrast (no filter) microscopy. Fluorescence of bacterial colonies on plates was examined with a LEICA Fluorescence Zoom Stereo Microscope. Images were captured at 7× magnification with a color camera.

## Results

### Objectives

Tag *B. anthracis, Y. pestis, B. mallei*, and *B. pseudomallei* surrogate genomes with constitutively expressed GFP/RFP in vitro and within macrophages to allow high-content analysis and screening of intracellular microbes. Proven constructs success will then allow engineering of their respective virulent select agent strains for commencement of compound and siRNA screens, and will serve as fluorescent background organisms for insertions of specific targetrons.

### Identification of constitutive and strong bacterial promoters

With the genetic tools that are available for engineering, the select agents *B. anthracis* Ames, *Y*. *pestis* CO92, *B*. *pseudomallei* K96243, *B*. *mallei* NBL7, and their respective surrogate strains *B. anthracis* Sterne, *Y. pseudotuberculosis*, and *B. thailandensis* (Choi et al. 2005; Janes and Stibitz 2006; Norris et al. 2009) as well as the ability to construct stable, constitutively expressed, chromosomal fluorescent transcriptional fusions, we first required the identification of constitutive and potent bacterial promoters of the aforementioned strains. We would expect such promoters to (i) drive maximum expression of the fusion reporters, (ii) such expression would not be deleterious to the bacterial host, and (iii) do not affect the genetic stability or virulence of such organism. To achieve this goal, we combined extensive literature searches and bioinformatic analyses for specific promoter identification in each of the aforementioned organisms. The promoter candidates were screened for expression of GFP in *E. coli* and the surrogate strains first and those with the strongest signal were then tested for activity in the surrogate and select agent strains.

### Genomic integrants marked with “Superfolder” green and TurboRed fluorescent proteins

#### *Bacillus anthracis* Ames strains (select agent) and *B. anthracis* Sterne (surrogate)

Gat et al. (2003) reported the most potent *B. anthracis* promoter, P*ntr*, which was isolated by the use of a promoter trap system via screening of a chromosomal-DNA library of *B. anthracis* fused to the fluorescent biotracer GFP. The 271-bp P*ntr* was found to be 500 times more potent than the native *pagA* promoter and 70 times more potent than the *α*-amylase promoter (P*amy*). Thus, the promoter P*ntr* was selected for the present study. In addition, Bergman et al. (2007) reported a genome-wide analysis of *B. anthracis* gene expression during infection of host phagocytes, and used custom *B. anthracis* microarrays to characterize the expression patterns occurring within intracellular bacteria throughout infection of the phagocytes. Hence, the highly informative microarray data described in the literature were screened using a bioinformatic approach for the top ∼3% of highly expressed genes (Table S1). Of these 11 genes that were invariant across all conditions tested were chosen for further analysis. We cloned Superfolder GFP under the control of promoters of GBAA_0253, GBAA_4533, and GBAA_5722, respectively; the promoter of GBAA 0253 (P0253)-driven GFP revealed bright and consistent fluorescence in host strains and was, therefore, selected for further study (Fig.2 and data not shown).

**Figure 1:**
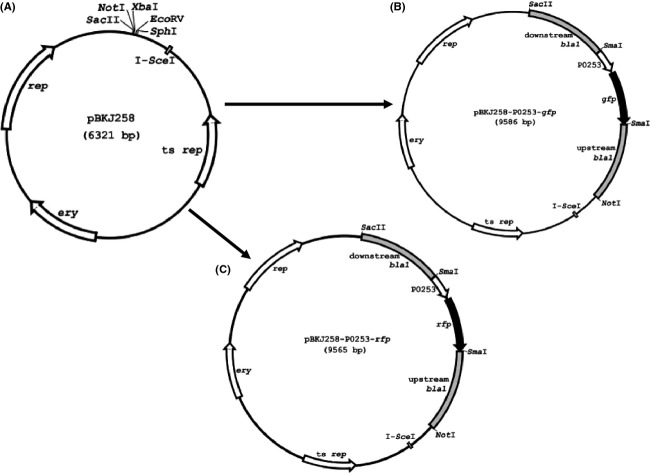
(A) Plasmid maps of pBKJ258, (B) pBK258-P0253-*gfp*, and (C) pBK258-P0253-*rfp*, respectively.

**Figure 2:**
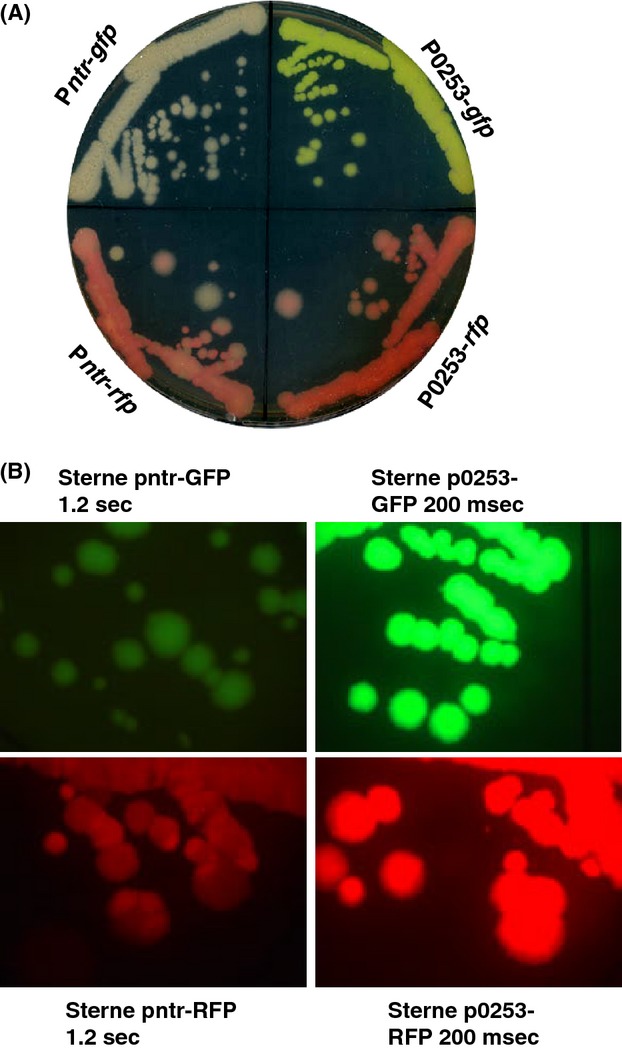
(A) Chromosomally tagged *Bacillus anthracis* Sterne strains were streaked out on a BHI agar plate. The plate was incubated at 37°C for 24 h and recorded by scanning. The bacteria in each quadrant were Sterne::P*ntr*-*gfp* (top left), Sterne::P*ntr*-*rfp* (bottom left), Sterne::P0253-*gfp* (top right), and Sterne::P0253-*rfp* (bottom right). (B) Fluorescence microscopy of bacteria in each quadrant on the same plate described in (A). Fluorescence images were taken with an exposure time of 1.2 sec for the strains Sterne::P*ntr*-*gfp* (top left) and Sterne::P*ntr*-*rfp* (bottom left), and with an exposure time of 200 msec for the strains Sterne::P0253-*gfp* (top right) and Sterne::P0253-*rfp* (bottom right).

We elected to use the allelic-exchange plasmid, pBKJ258, and helper plasmid, pBKJ223, developed by Janes and Stibitz (2006) and Lee et al. (2007) for chromosomal tagging of *B. anthracis* with GFP and RFP reporter fusions. The advantage of such a system was that it generated markerless mutations in *B. anthracis* that facilitated the downstream genetic manipulation with the reporter-tagged strains, especially the select agent *B. anthracis* Ames, which has very few approved antibiotics as selection markers. To generate a gene replacement construct for integration into the *B. anthracis* chromosome by homologous recombination, ∼1 kb of upstream and downstream fragments of the *bla*1 gene (GBAA_2507, encoding *β*-lactamase) was cloned between the *Not*I and *Sac*II sites of pBKJ258 (Fig.1A) (Janes and Stibitz 2006), creating pBKJ258Δ*bla1*. The p0253 promoter (or P*ntr* promoter)-driven Superfolder *gfp* or TurboRed *rfp* were then inserted into the unique *Sma*I site generated at the juncture of flanking sequences of the *bla1* gene within pBKJΔ*bla1*. The resulting reporter constructs, pBKJ258-P*ntr*-*gfp*, pBKJ258-P*ntr*-*rfp*, pBKJ258-P0253-*gfp* (Fig.1B), and pBKJ258-P0253-*rfp* (Fig.1C) were confirmed by DNA sequencing. We next introduced the above constructs into *B. anthracis* Sterne via conjugation or electroporation at room temperature. Plasmid integrants were isolated by a shift to the replication-nonpermissive temperature while maintaining selection for erythromycin resistance. The double crossover, homologous recombination event was achieved by the introduction of a second plasmid, pBKJ223, which was then lost spontaneously following screening by PCR and DNA sequencing for the desired replacement of the *bla1* gene with P0253 (or P*ntr*)-driven *gfp* or *rfp* reporter fusions. The allelic-exchange procedure performed here has previously been described in detail (Janes and Stibitz 2006). The chromosomal GFP- and RFP-tagged *B. anthracis* Sterne strains were streaked out on BHI agar plates and examined by fluorescence microscopy. As shown in Figure2A, the greenish or reddish colonies of GFP- or RFP-tagged bacteria appeared on BHI agar and could be observed very easily with the naked eye. However, the bacteria tagged with P*ntr*-*gfp* or P*ntr*-*rfp* emitted weaker fluorescence (Fig.2A, top and bottom left quadrants) than bacteria tagged with P0253-*gfp* or P0253-*rfp* (Fig.2A, top and bottom right quadrants). As expected, all bacterial cultures streaked on the same plate were fluorescent using fluorescence microscopy (Fig.2B). *Bacillus anthracis* Sterne tagged with P0253-*gfp* or P0253-*rfp* exhibited a much brighter fluorescent signal, even with an exposure time of 200 msec than bacteria marked with P*ntr*-*gfp* or P*ntr*-*rfp* with an exposure time of 1.2 sec (Fig.2B), indicating that the P0253 promoter was much stronger than that of P*ntr* in *B. anthracis*. Therefore, we selected the P0253 promoter-driven reporters only for tagging the Ames select agent strain.

**Figure 3:**
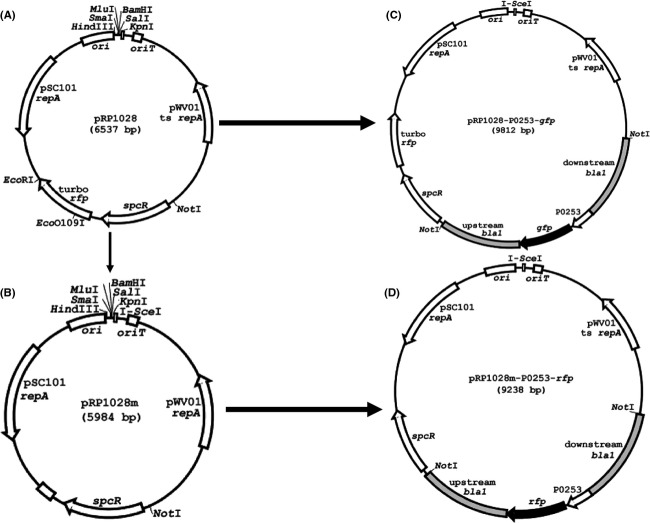
(A) Plasmid maps of pRP1028, (B) pRP1028m, (C) pRP1028-P0253-*gfp*, and (D) pRP1028m-P0253-*rfp*, respectively.

**Figure 4:**
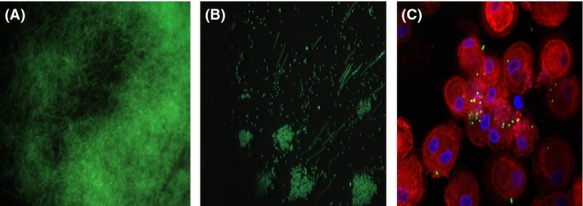
Fluorescence micrographs of (A) *Bacillus anthracis* vegetative cells, (B) *B. anthracis* spores, and (C) *B. anthracis* spore expressing GFP within monocyte-derived macrophages.

Since the erythromycin resistance marker (*ery*) in pBKJ258 and the tetracycline resistance marker (*tet*) in pBKJ223 were not approved for use in the select agent *B. anthracis* Ames strain, we acquired another gene replacement plasmid, pRP1028 (Fig.3A), and helper plasmid, pSS4332. Plasmids pRP1028 and pSS4332 were used as improvements to the previously published system (Janes and Stibitz 2006), and served the same functions of pBKJ258 and pBKJ223, respectively. Plasmid pRP1028 carries a spectinomycin resistance selectable marker (*spcR*) and pSS4332 harbors a kanamycin resistance selectable marker (*kan*), respectively. Both antibiotic resistance markers were appropriate for use in select agent strains. Considering that the presence of an *rfp* gene in the plasmid backbone of pRP1028 (a feature facilitating a direct visual screen for plasmid loss following introduction of pSS4332 to promote recombination) could interfere with the RFP tagging of the Ames strain, we modified pRP1028 by *Eco*RI and *Eco*OI091 digestion to remove 552 bp internal sequence of the *turbo rfp* gene, thereby creating pRP1028m (Fig.3B). Next, a 3029-bp fragment containing Δ*bla1*::P0253-*gfp* or -*rfp* from pBKJ258-P0253-*gfp* (Fig.1B) and pBKJ258-P0253-*rfp* (Fig.1C) was PCR amplified and cloned into the unique *Not*I site of pRP1028 and pRP1028m, respectively. The resultant allelic-exchange reporter constructs, pRP1028-P0253-*gfp* (Fig.3C) and pRP1028-P0253-*rfp* (Fig.3D), were verified by DNA sequencing and introduced into the Ames strain. Plasmid integrants were isolated following a temperature shift while maintaining selection for spectinomycin resistance (Janes and Stibitz 2006). Introduction of pSS4332 into the integrant led to I-*Sce*I-mediated cleavage of the integrated plasmid, stimulating the second crossover event. Loss of all engineered plasmids used for chromosomal integration was demonstrated by a concomitant loss of antibiotic resistance. The presence of the virulence plasmids pXO1 and pXO2, and the desired chromosomal replacement of *bla1* gene with P0253-*gfp* or *-rfp* reporter fusions in the Ames strain was confirmed by PCR and sequencing. The GFP- and RFP-marked Ames strains were finally validated by fluorescence microscopy. Figure4 showed the fluorescence microscopic analyses of vegetative cells (Fig.4A) and spores (Fig.4B) of the GFP-tagged *B. anthracis* Ames as well as THP-1 macrophages infected with GFP-tagged spores derived from the Ames strain (Fig.4C).

#### *Yersina pestis* strains CO92 (select agent) and *Yersina pseudotuberculosis* (surrogate)

Bland et al. (2011) identified a strong, likely constitutive promoter, P*cysZK*, in *Y. pestis* by screening a library of *Y. pestis* KIM D27 DNA fragments fused to a promoterless DsRed. P*cysZK* was reported to drive expression of the fluorescent protein in laboratory media or during macrophage infection, permitting detection by confocal laser scanning microscopy in single copy. Therefore, P*cysZK* was chosen as the primary strong promoter candidate for the *Yersinia* strains. We also analyzed the whole-genome microarray data of the *Y. pestis* in vivo transcriptome in infected fleas reported by Vadyvaloo et al. (2010), and categorized *Y. pestis* promoters into three promoter expression groups: 54 low, 36 medium, and 48 high (Table S2). We chose six promoters (P*rplJ*(YPO3749), P*relC*(YPO3751), P*rplN*(YPO0220), P*nusE*(YPO0209), P*rpsM*(YPO0231), and P*rplU*(YPO3712)) as a high-expression group along with P*cysZK* for promoter activity studies.

**Figure 5:**
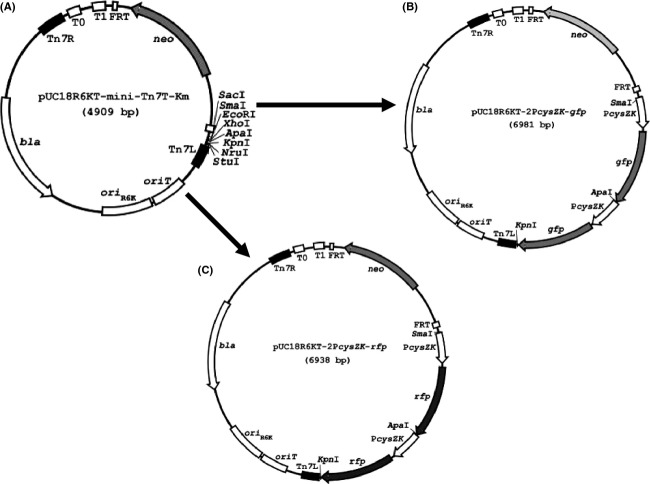
Plasmid maps of (A) pUC18R6KT-mini-Tn*7*T-Km, (B) pUC18R6KT-2P*cysZK-gfp*, and (C) pUC18R6KT-2P*cysZK-rfp*, respectively.

**Figure 6:**
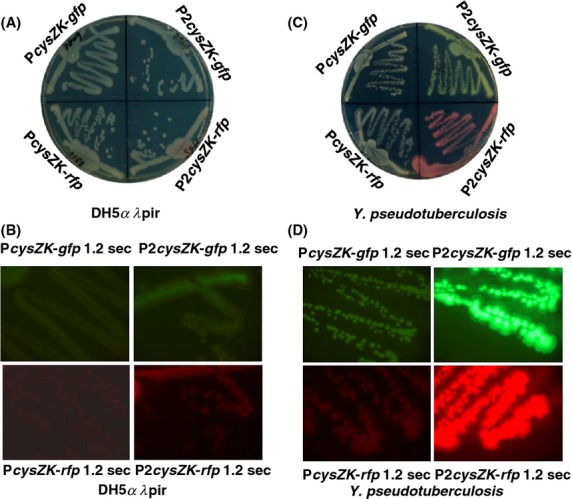
(A) *Escherichia coli* DH5*α λpir* strains harboring reporter plasmid construct pUC18R6KT-P*cysZK-gfp*, pUC18R6KT-P*cysZK-rfp*, pUC18R6KT-2P*cysZK-gfp*, or pUC18R6KT-2P*cysZK-rfp* were struck on LB-agar plates. (B) Colony fluorescence microscopy of bacteria in each quadrant on the same plate described in (A). (C) Chromosomally integrated *Yersina pseudotuberculosis* strains were also struck out on L-agar plates and colony micrographs demonstrating GFP and RFP fluorescence are shown in (D).

Next, we elected to tag *Y. pseudotuberculosis* and *Y. pestis* using a Tn*7*-based, broad-range bacterial cloning and expression system reported by Choi et al. (2005). The system, consisting of a mini-Tn*7* vector and a helper plasmid pTNS2 encoding the site-specific TnsABC+D transposition pathway, allows the engineering of diverse genetic traits into bacteria, including *Y. pestis*, at a single *att*Tn*7* site downstream of the *glmS* gene (Choi et al. 2005). The *Yersinia* promoters p*cysZK*, P*rplJ*, P*relC*, P*rplN*, P*nusE*, P*rpsM*, and P*rplU* were first PCR amplified using genomic DNA of *Y. pestis* CO92 as the template, restricted with *Sma*I and *Pst*I, and ligated with Superfolder *gfp* or TurboRed *rfp* digested with *Pst*I and *Apa*I. The reporter fusions were then cloned between *Sma*I and *Apa*I sites of the mini-Tn7 vector, pUC18R6KT-mini-Tn*7*T-Km (Fig.5A), respectively. The resultant constructs were confirmed by DNA sequencing and mobilized into *Y. pseudotuberculosis* by triparental mating using *E. coli* conjugation strain RHO3 (Lopez et al. 2009) and helper plasmid pTNS2. Kanamycin-resistant conjugants with chromosomal Tn*7* insertions in *Y. pseudotuberculosis* were verified by PCR analysis using primer pair YPatt5′ and YPatt3′, and primer pair PTn*7*L and PTn*7*R. The kanamycin resistance marker was removed by Flp recombinase-mediated excision via plasmid pFLP2, which was then cured by sucrose counterselection. All GFP- and RFP-tagged *Y. pseudotuberculosis* strains were examined by fluorescence microscopy. Unexpectedly, strains marked with P*rplJ*-, P*relC*-, P*rplN*-, P*nusE*-, P*rpsM*-, or P*rplU*-driven reporter fusions exhibited very weak fluorescent signals (data not shown) and were therefore not pursued further. Consistent with the previous study (Bland et al. 2011), P*cysZK* showed strong promoter strength in *Yersinia* strains. As shown in Figure6, *E. coli* DH5*α λ*pir harboring mini-Tn*7* construct (pUC18R6KT-P*cysZK*-*gfp*) or (pUC18R6KT-P*cysZK*-*rfp*) grown on LB plates (Fig.6A, top and bottom left quadrants) displayed fluorescent signals using fluorescence microscopy with an exposure time of 1.2 sec (Fig.6B, top and bottom left quadrants). In contrast, the chromosomal P*cysZK*-*gfp-* or P*cysZK*-*rfp-*tagged *Y. pseudotuberculosis* strains cultured on LB plates (Fig.6C, top and bottom left quadrants) exhibited brighter fluorescence (Fig.6D, top and bottom left quadrants) than DH5*α λ*pir (pUC18R6KT-P*cysZK*-*gfp*) or DH5*α λ*pir (pUC18R6KT-P*cysZK*-*rfp*), respectively. Considering the presence of multiple copies of plasmid in *E. coli*, P*cysZK* was much more active in *Yersinia* strains.

**Figure 7:**
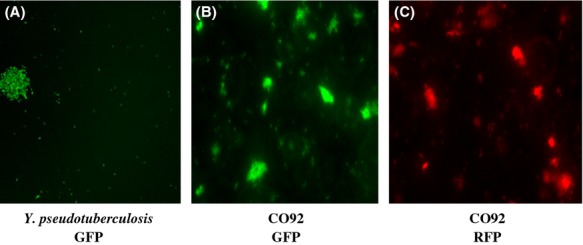
Fluorescence micrographs of (A) *Yersina pseudotuberculosis* GFP, (B) *Y. pestis* CO92 GFP, and (C) *Y. pestis* CO92 RFP strains with the fusions inserted into the chromosomal Tn*7* site.

The success of tagging surrogate and fluorescent *Y. pseudotuberculosis* next led us to the goal of marking the virulent select agent strain, *Y. pestis* CO92, with P*cysZK*-*gfp* and P*cysZK*-*rfp* following the identical procedure described above. The tagged CO92 strain fluoresced well originally with an exposure time of 200 msec. Unfortunately, the fluorescent signal of the tagged strain became dimmer in subsequent experiments and thus was deemed inappropriate for HTS analyses. Hence, we elected to enhance fluorescence by tagging *Yersinia* strains chromosomally with two copies of the fusions. Therefore, a second copy of P*cysZK*-*gfp* or P*cysZK*-*rfp* was cloned between the *Apa*I and *Kpn*I sites of pUC18R6KT-P*cysZK*-*gfp* or pUC18R6KT-P*cysZK*-*rfp* in the same orientation of the first copy of the fusion, creating pUC18R6KT-2P*cysZK*-*gfp* (Fig.5B) and pUC18R6KT-2P*cysZK*-*rfp* (Fig.5C), respectively. Following the same steps described above, two copies of reporter fusions 2P*cysZK*-*gfp* and 2P*cysZK*-*rfp* were successfully inserted at the chromosomal Tn*7* site of surrogate strain *Y. pseudotuberculosis* and the select agent, *Y. pestis* CO92. The resultant strains were then validated by fluorescence microscopy. The difference between one copy of each reporter construct versus two copies were apparent in terms of the fluorescence signal emitted as demonstrated in Figure6. *Escherichia coli* DH5*α λ*pir(pUC18R6KT-2P*cysZK*-*gfp*) or DH5*α λ*pir(pUC18R6KT-2P*cysZK*-*rfp*) cultured on an LB plate (Fig.6A, top and bottom right quadrants) emitted brighter fluorescence (Fig.6B, top and bottom right quadrants) than DH5*α λ*pir(pUC18R6KT-P*cysZK*-*gfp*) or DH5*α λ*pir(pUC18R6KT-P*cysZK*-*rfp*) (Fig.6A and B, top and bottom left quadrants) with the same exposure time. As expected, *Y. pseudotuberculosis* tagged with 2P*cysZK*-*gfp* or 2P*cysZK*-*rfp* reporters produced greenish or reddish colonies on LB plates (Fig.6C, top and bottom right quadrants) and yielded a far brighter fluorescence signal (Fig.6D, top and bottom right quadrants) than *Y. pseudotuberculosis* tagged with one copy of the reporter fusions (Fig.6C and D, top and bottom left quadrants). Bacterial cells of 2P*cysZK*-*gfp*- or 2P*cysZK*-*rfp*-tagged *Y. pseudotuberculosis* and *Y. pestis* CO92 from planktonic cultures fluoresced well with an exposure time of 200 msec (Fig.7A–C). In addition, the presence of three important natural virulence plasmids pCP1, pMT1, and pCD1 in the tagged CO92 strains were confirmed by PCR analysis with primer pair Pla5′ and Pla3′ (specific to *pla* gene in pCP1, encoding the plasminogen activator; McDonough and Falkow 1989), Ymt5′ and Ymt3′ (specific to *ymt* gene in pMT1, encoding a murine toxin phospholipase D; Rudolph et al. 1999), and LcrV5′ and LcrV3′ (specific to *lcrV* gene in pCD1, encoding a major protective antigen; Titball and Williamson 2001) (data not shown).

#### *Burkholderia mallei* NBL and *pseudomallei* strains K96243 (select agent) and *B. thailandensis* (surrogate)

Yu and Tsang (2006) described the use of the ribosomal *rpsL* promoter PC_*S12*_ from *Burkholderia cenocepacia* LMG16656 and from *B. cepacia* MBA4 for efficient expression of a functional transporter protein in *E. coli*. Norris et al. (2010) utilized the enhanced GFP (eGFP) and other optimized fluorescent protein genes driven by the *rpsL* promoter P_*S12*_ of *B. pseudomallei* to achieve stable, site-specific fluorescent labeling of *B. pseudomallei* and *B. thailandensis*. A constitutive broad-host-range P1 integron promoter showed potency to drive the expression of Tn*7*-site-specific transposase and the *lux* operon for bioluminescent imaging in *Burkholderia* spp. (Choi et al. 2008; Massey et al. 2011). Therefore, we selected P_*S12*_ and P1 as good promoter candidates for further study. In addition, based on a proteome reference map and protein abundance of *B. pseudomallei* during the stationary growth phase (Wongtrakoongate et al. 2007), promoters of genes encoding chaperonin GroES, the heat shock protein (GrpE) and phasin-like protein (PhaP), were also considered. Based on microarray data derived from *B. pseudomallei* K96243 (Rodrigues et al. 2006), our bioinformatic analysis predicted 63 genes that were highly expressed, which were also on the promoter candidate list (Table S3).

The use of specific selectable antibiotics resistance cassettes in both *B. pseudomallei* and *B. mallei* is strictly regulated. Only a few antibiotics, including gentamicin, kanamycin, and zeocin, are currently approved for use in these bacteria, but wild-type strains are highly resistant to these antibiotics (Schweizer and Peacock 2008). Norris et al. (2009) reported glyphosate (the active ingredient in the herbicide, Round-Up™) resistance as a novel select-agent-compliant, nonantibiotic-selectable marker for *Burkholderia* select-agent species, and developed several mini-Tn*7* vectors for stable, site-specific fluorescent tagging of *B. pseudomallei* and *B. thailandensis* based on the *gat* gene, which encodes glyphosate acetyltransferase and confers resistance to the common herbicide, glyphosate (Norris et al. 2010). Hence, we elected to use *gat* as a selectable marker and chose the site-specific transposon pmini-Tn*7*-*gat* (Norris et al. 2009, Fig.8A) as the vector to deliver the reporter fusions into the genomes of *Burkholderia* spp. Since the constructs mini-Tn*7*-*gat-gfp* and mini-Tn*7*-*gat-rfp*, in which both e*gfp* and optimized *rfp* were driven by the P_*S12*_ promoter, and the relevant *E. coli* strains for fluorescent labeling of *Burkholderia* spp. were already available, we tagged *B. thailandensis* with P_*S12*_-*gfp* and P_*S12*_-*rfp* at the chromosomal *att*Tn*7* sites using the above constructs following previously described procedures (Norris et al. 2010). The tagged surrogates fluoresced with an exposure time of 2 sec, but the fluorescent signal was not detectable with an exposure time of 200 msec (data not shown). Thus, the reporter fusions P_*S12*_-*gfp* and P_*S12*_-*rfp* were not suitable for high-throughput screening. Therefore, for further studies to proceed, a search for promoters stronger than P_*S12*_ in *Burkholderia* spp. was required.

**Figure 8:**
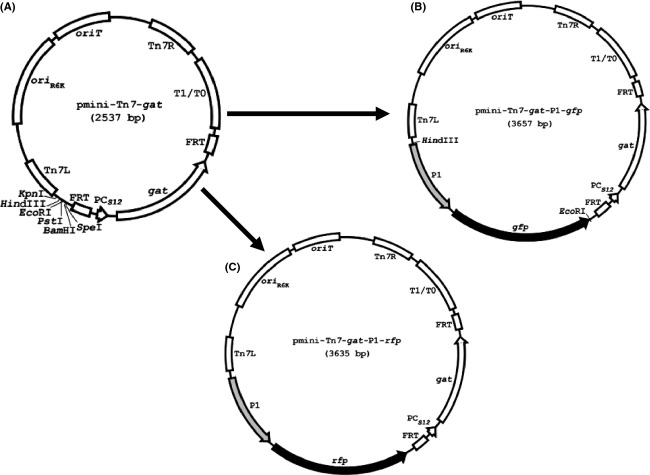
Plasmids maps of (A) pmini-Tn*7*-*gat*, (B) pmini-Tn*7*-*gat*-P1-*gfp*, and (C) pmini-Tn*7*-*gat*-P1-*rfp*, respectively.

**Figure 9:**
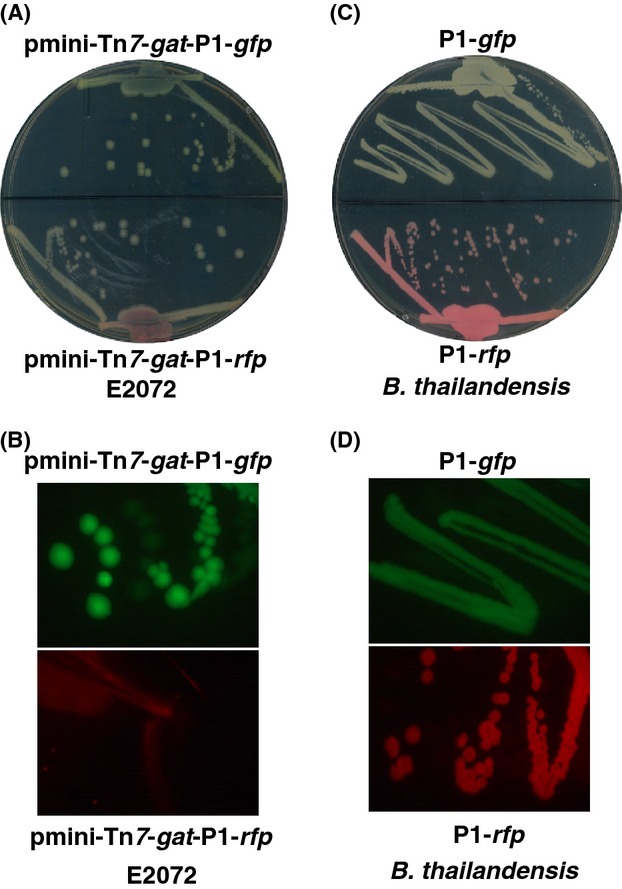
(A) *Escherichia coli* E2072 (pmini-Tn*7*-*gat*-P1-*gfp*) (top of plate) and *E. coli* E2072(pmini-Tn*7*-*gat*-P1-*rfp*) (bottom of plate) struck out on LB-agar plates. (B) Fluorescence microscopy of the *E. coli* strains described in (A). (C) Chromosomal *glmS1* att-Tn*7* site-integrated *B. thailandensis* Tn*7*-P1-*gfp* (top of plate) and *B. thailandensis* Tn*7*-P1-*rfp* (bottom of plate) that struck out on LB-agar plates. (D) Fluorescence microscopy of the *B. thailandensis* strains described in (C).

**Figure 10:**
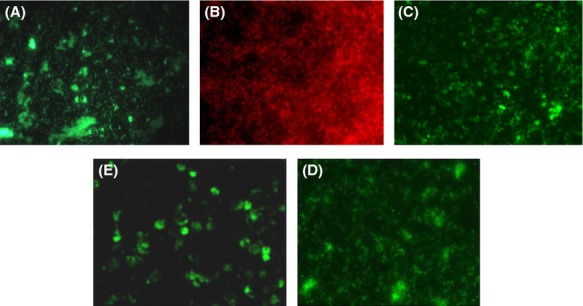
Fluorescence microscopy of chromosomal *glmS1* att-Tn*7* site-integrated *Burkholderia* GFP and RFP fusion strains. (A) *Burkholderia thailandensis* GFP, (B) *B. thailandensis* RFP, (C) *B. mallei* GFP, (D) *B. pseudomallei* GFP, and (E) *B. mallei* expressing GFP within THP-1 macrophages.

Next, *Burkholderia* promoters P*groES*, P*grpE*, and P*phap* and the integron promoter P1 were assayed for their strength to drive the expression of each reporter. Specifically, promoters P*groES*, P*grpE* (GrpE), and P*phap* were PCR amplified using genomic DNA of *B. pseudomallei* as templates. Promoter P1 was amplified by PCR using pTNS3-*asd*_*Ec*_ as template. The reporters *egfp* and the optimized *rfp* for *Burkholderia spp*. were PCR amplified from mini-Tn*7*-*gat-gfp* and mini-Tn*7*-*gat-rfp*, respectively. All amplified promoter fragments were digested with *Hind*III and *Pst*I, and ligated with *Pst*I and *Eco*RI*-*restricted *egfp* and *rfp*. The promoter-reporter fusions were then cloned between *Hind*III and *Eco*RI sites of pmini-Tn*7*-*gat* (Fig.8A). The resultant constructs, pmini-Tn*7*-*gat-*P1-*gfp* (Fig.8B), pmini-Tn*7*-*gat*-P1-*rfp* (Fig.8C), and others were confirmed by DNA sequencing and transformed into the *E. coli* donor strain, E2072. Each fluorescent tag was then introduced into the surrogate strain *B. thailandensis* by triparental mating using *E. coli* helper strain E1354 (pTNS3-*asd*_*Ec*_) as previously described (Norris et al. 2009). *Burkholderia thailandensis* containing the inserted transposon was selected on 1× M9 minimal glucose medium containing 0.3% glyphosate. Insertion at the chromosomal *glmS1* or *glmS2 att*-Tn7 sites of *B. thailandensis* was verified by PCR as previously described (Choi et al. 2008; Kang et al. 2009; Norris et al. 2009), utilizing two primers BTglmS1 and BTglmS2 in combination with primer Tn*7*L. All tagged strains with insertion at *glmS1 att*-Tn7 site were then examined by fluorescence microscopy. *Burkholderia thailandensis* labeled with P*groES*-, P*grpE*-, P*phap*-*gfp*, or *-rfp* produced very weak fluorescent signals (data not shown) and thus were not used for the further study. In contrast, the P1 integron promoter demonstrated much stronger promoter activity to drive the expression of fluorescent proteins in both *E. coli* and *B. thailandensis*. As shown in Figure9, *E. coli* E2072 harboring plasmid construct pmini-Tn*7*-*gat-*P1-*gfp* or pmini-Tn*7*-*gat-*P1-*rfp* struck on an LB plate (Fig.9A) displayed fluorescent signal using low-magnification microscopy (Fig.9B). *Burkholderia thailandensis* labeled with P1-*gfp* or P1-*rfp* produced greenish or reddish colonies on an LB plate (Fig.9C), and exhibited a bright fluorescent signal with an exposure time of 200 msec (Fig.9D, 10A and B). As the surrogate strain *B. thailandensis* was successfully labeled with P1-*gfp* or P1-*rfp*, and yielded a high fluorescent signal, we subsequently tagged the select agents *B. mallei* NBL and *B. pseudomallei* strain K96243 genomes with P1-*gfp* or P1-*rfp* following the same procedure as described above. The genomic DNA of both GFP- and RFP-tagged select agent strains was isolated and PCR analysis using the following primers confirmed the reporter insertion at the chromosomal *att-*Tn*7* sites as previously described (Choi et al. 2006, 2008; Norris et al. 2009). For *B. mallei*, the two PCR primers BMglmS1 and BMglmS2 were used to determine Tn*7* insertion downstream of either *glmS1* or *glmS2* in combination with primer Tn*7*L. For *B. pseudomallei*, insertions at the *glmS1, glmS2*, and *glmS3 att-*Tn*7* sites were verified utilizing primers BPglmS1, BPglmS2, and BPglmS3 in combination with primer Tn7L. Examination by fluorescence microscopy demonstrated that *B. pseudomallei* and *B. mallei* cells tagged with P1-*gfp* or P1-*rfp* at the *glmS1 att-*Tn*7* sites generated bright fluorescent signals (Fig.10 B–D and data not shown).

#### Bacterial growth and LDH release in macrophages upon infection with GFP-tagged *B. anthracis, Y. pestis, B. mallei*, and *B. pseudomallei*

Upon completion of all above fluorescent tagging work on these bacteria, we next used four GFP-tagged select agents, *B. anthracis* Ames, *Y. pestis* CO92, *B. mallei*, and *B. pseudomallei* to infect differentiated human monocytic THP-1 cells and performed image analysis, bacterial load determinations, and LDH assays over time. As shown in Figure1, compared to the initial bacterial load at the zero time point, no increase in CFU counts were observed for all strains within macrophages at 12 and 24 h postinfection. At 48 h postinfection, bacterial density increased only slightly. In contrast, CFU counts of all strains rose dramatically within macrophage at 72 h postinfection with nearly 20 times increase for *B. anthracis* Ames, 3.5 times for *Y. pestis* CO92, and 2–3 times for *B. pseudomallei* and *B. mallei*, respectively. In parallel, our LDH assays indicated that *Y. pestis-, B. mallei*-, and *B. pseudomallei*-infected macrophages retained 80% viability during the first 24 h postinfection, but dropped significantly to approximate 40% at 48 h postinfection. Viability continued to drop by 20% at 72 h postinfection (Fig.2). In contrast, the viability of *B. anthracis*-infected macrophages dropped continuously at each time point, albeit 20% viability was reached at 72 h postinfection (Fig.2). The above kinetics of bacterial propagation within macrophages and the release of LDH, a biomarker for cellular cytotoxicity and cytolysis, indicated that the engineered GFP tags were useful to track the select agents *B. anthracis* Ames, *Y. pestis* CO92, *B. mallei*, and *B. pseudomallei*, respectively.

**Figure 11:**
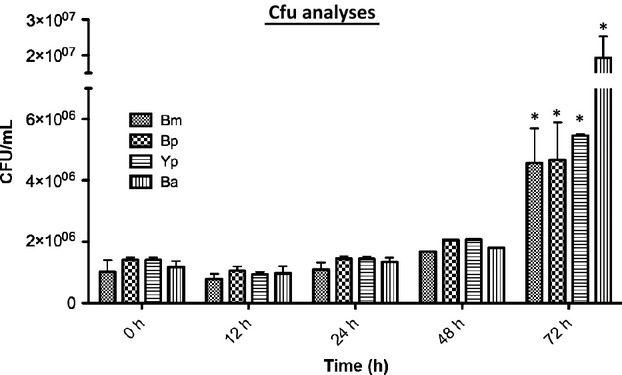
Time course of colony forming unit analysis of GFP-tagged *B. anthracis* (Ba), *Y. pestis* (Yp), *B. mallei* (Bm), and *B. pseudomallei* (Bp) after phagocytosis by THP-1 macrophages (*n* = 3). The stars (*) above the bars at the 72 h time point indicate a significance level of *P* < 0.05.

**Figure 12:**
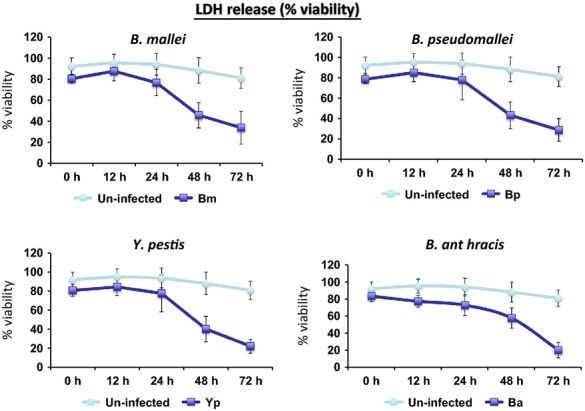
LDH release over a period of 3 days from THP-1 macrophages infected with GFP-tagged *B. anthracis, Y. pestis, B. mallei*, and *B. pseudomallei* (infected, dark blue line) versus uninfected (light blue line) (*n* = 3).

## Discussion

In this study, we constructed chromosomal, constitutively expressed GFP and RFP fusions in *B. anthracis* Ames, *Y. pestis, B. mallei, B. pseudomallei*, and their surrogates *B. anthracis* Sterne, *Y. pseudotuberculosis*, and *B. thailandensis*, respectively. Considering the potential use of these tagged strains in large-scale high-throughput chemical compound screening, siRNA screening, genome-wide mutant construction and subsequent macrophage survival assays, the fluorescent strains were anticipated to meet the following criteria with regard to the GFP or RFP reporter (i) *in cis*, chromosomal integration, (ii) stable maintenance, (iii) constitutive expression, (iv) wild-type virulence fitness, and (v) less than 200 msec exposure time required in a high-throughput chemical screening analyses. This last feature (v) is likely the most relevant for future use and was the greatest challenge, since the equipment used for our fluorescent measurements required <200 msec exposure, and none of the reported GFP and RFP reporters at the single-copy level can meet such a short exposure time requirement. Thus, we present here the demonstration of the completion of this challenging genomic tagging work in the aforementioned highly virulent strains and their surrogates. Due to the strict requirements and high expenses for handling the select agents, all reporter fusions were validated in their respective surrogates prior to delivery into the human-virulent, select agent strains. The respective GFP and RFP tagging would facilitate bacterial coinfection or competitive infection assays where two strains/mutants expressing different colors could be located or tracked.

To achieve our goal of obtaining stable, chromosomal, constitutive GFP/RFP reporters in these highly virulent select agents and their surrogates, we first needed to select *gfp* and *rfp* reporter genes adapted to use in the aforementioned organisms. The wild-type *gfp* and *rfp* have been mutated or optimized based on codon usage preferred by bacteria to improve detection and expression of the fluorescent protein in prokaryotes (Heim et al. 1995; Cormack et al. 1996; Miller and Lindow 1997; Norris et al. 2010). We chose the GFP Superfolder and RFP TurboRed (Su et al. 2013) as fluorescent proteins for *B. anthracis, Y. pestis*, and *Y. pseudotuberculosis*, and the eGFP and optimized RFP (Norris et al. 2010) for *B. mallei* and *B. pseudomallei*, which have been utilized successfully in fluorescent tagging of *Francisella* spp. (Su et al. 2013) and *Burkholderia* spp. (Norris et al. 2010). Second, a bioinformatic approach coupled with exhaustive literature searches were used to predict the strongest, constitutive bacterial promoters suitable for our purposes. Two *B. anthracis* promoters were selected based on both techniques and preliminary screening. The first, P0253 was rated “hot and invariant” using the bioinformatic search, a rating that was considered the highest possible using the programed algorithm (see Materials and Methods section and Table S1). The second, P*ntr*, was identified by a promoter trap system to be most potent *B. anthracis* promoter, 10 times more potent than the very strong P*saplong* promoter and 70 times more efficient than P*amy* in eliciting GFP expression in *B. anthracis* (Gat et al. 2003). While both promoters were capable of driving expression of *gfp* and *rfp* at the single copy in the surrogate strain *B. anthracis* Sterne, P0253 exhibited much more stronger promoter strength than P*ntr* by approximately 30-fold as demonstrated by the fluorescence intensity (Fig.3). Hence, only P0253 was selected for use in *B. anthracis* Ames, and its fluorescence in the select agent was indistinguishable from that of its surrogate strain. Fortunately, both vegetative cells and spores of *B. anthracis* were fluorescently tagged (Fig.4), indicating the promoter P0253 was continuously on from vegetative growth phase to the sporulation stages, in all cell forms of the organism. P*ntr* is a nitroreductase promoter (Gat et al. 2003). P0253 was located at the upstream promoter region of a gene (GBAA_0253) encoding a hypothetical protein comprising of 95 amino acids. The protein could be one of the most abundant proteins in the organism because the promoter P0253 was constitutive and robust, most likely the strongest promoter in *B. anthracis* so far to the best of our knowledge. It is noteworthy that characterization of function of the gene (GBAA_0253) should be performed for future studies.

Choi et al. (2005) described “GFP tagging” *Y. pestis* by utilizing a mini-Tn*7* construct expressing GFP from the *neo* promoter (P*neo*) followed integration into the *Y. pestis* A1122 chromosome, and determined the distribution of GFP-expressing bacteria within mouse peritoneal exudate cells. However, the fluorescent signal delivered by such a reporter fusion was too weak to be detectable with an exposure time of 200 msec (data not shown). In agreement with the report by Bland et al. (2011), our study also showed that promoter P*cysZK* (YPO2992) supported the highest level of fluorescence expression in single copy in *Y. pestis. cysZ* is a conserved, nonessential gene that encodes an inner membrane sulfate transporter protein (Parra et al. 1983), which would be highly abundant as this is a key nutrient for cells during all phases of growth (Bland et al. 2011). Despite the fact that the promoter P*cysZK* was not a “hit” in our bioinformatics search, it displayed experimentally much stronger promoter activity to drive the reporter expression than those predicted to be high, including as P*rplJ*-, P*relC*-, P*rplN*-, P*nusE*-, P*rpsM*-, or P*rplU*. Unexpectedly, we observed fluorescence instability with P*cysZK-gfp/rfp-*tagged *Y. pestis*, which fluoresced brighter initially but the fluorescent signal became weaker in the subsequent experiments. We reasoned a mutation might occur in either the promoter or the reporter gene during bacterial propagation after integration of the reporter fusion into the genome, but sequencing analysis ruled out this possibility. Introduction of a second copy of the P*cysZK-gfp/rfp* reporter fusion into the *Yersinia* genome not only stabilized the fluorescence but also significantly increased the fluorescence signal (Fig.6).

Norris et al. (2010) constructed and demonstrated the use of several vectors for stable and site-specific fluorescent tagging of *B. pseudomallei* and *B. thailandensis*. These tools employed the *rpsL* promoter P_*S12*_ to drive the expression of *egfp* and other optimized fluorescent protein genes (cyan, red, and yellow) to achieve chromosomal labeling of *Burkholderia* spp. (Norris et al. 2010). However, the fluorescent signal intensity of the above chromosomal fusions P_*S12*_-*egfp* and P_*S12*_-*rfp* in *B. thailandensis* was not high enough to be detected with an exposure time of 200 msec, neither were the fusions P*groES*-, P*grpE*-, P*phap*-*gfp, or* -*rfp* (data not shown). During the screening for other potent promoter candidates, the P1 integron promoter-driven reporter fusions yielded the strongest fluorescence in single copy in the surrogate strain with an exposure time of 200 msec and was further demonstrated in *B. pseudomallei* and *B. mallei* (Figs. 9, 10). The relative strength of P1 integron promoter in plasmid R388 and in transposon Tn*1696* was first characterized to be six times more efficient in *E. coli* than the depressed *tac* promoter (Levesque et al. 1994). The P1 integron promoter was mostly associated with antibiotic resistance cassettes naturally and was also shown to be active in *Burkholderia* spp. It had been cloned and used as a common promoter to drive the expression of antibiotic-resistant elements or transposase in many broad-host-range vectors (DeShazer and Woods 1996; Choi et al. 2008). Unexpectedly, in this study, we revealed that P1 was the strongest exogenous promoter in *B. thailandesis, B. mallei*, and *B. pseudomallei* to date.

When applicable, the mini-Tn*7* transposon system is a convenient and most efficient delivery tool for site-specific genomic tagging of bacteria in which the tagging DNA is stably inserted at a unique and neutral chromosomal site. So far we have utilized different vectors based on such system to make the fluorescent labeling in gram-negative bacteria *F. tularensis* Schu S4 and LVS (Su et al. 2013), *Y. pestis, Y. pseudotuberculosis, B. thailandensis, B. pseudomallei*, and *B. mallei*. Despite the Tn7 transposition has not yet been documented in gram-positive bacteria, the markerless allelic exchange system developed by Janes and Stibitz (2006) also worked efficiently in tagging both Sterne and Ames strains of *B. anthracis*, a gram-positive bacteria. As the transcription of the *bla1* gene encoding the *β*-lactamase was silent in the Sterne strain (Chen et al. 2003) coupled with our anticipation of using *β*-lactam antibiotics as a potential selectable marker, we chose the *bla1* locus as the gene replacement target for fluorescent tagging of the *B. anthracis*.

In summary, what is the importance and utility of such GFP or RFP constitutively expressed strains? Undoubtedly, they will be beneficial for fundamental microbiological studies and can be applied to a variety of important and very revealing types of new and important information. These include (i) high-throughput compound screening (especially requiring at least a 200 msec time frame for HTS microscopy), (ii) virulence studies, (iii) vaccine development, (iv) in vivo animal models, (v) infective indices experiments, (vi) intramacrophage survival studies, as well as (vii) siRNA screening.

## Supporting Information

**Table S1.** Genes of *Bacillus anthracis* Ames identified by bioinformatic analysis to be expressed at high, medium, or moderately low levels with minimal variation during culture or macrophage infection. The analysis was based on affymetrix data sets http://www.ebi.ac.uk/arrayexpress/ (accession number E-MEXP-1036) (Bergman et al. 2007), http://www.ncbi.nlm.nih.gov/geo/query/acc.cgi?acc=GSE840 (Liu et al. 2004), and http://www.ncbi.nlm.nih.gov/geo/query/acc.cgi?acc=GPL370. See Materials and Methods section for details.

**Table S2.** Genes of *Yersinia pestis* CO92 identified by bioinformatic analysis to be expressed at high, medium, or moderately low levels with minimal variation during culture or macrophage infection. The analysis was based on affymetrix data sets http://www.ncbi.nlm.nih.gov/geo/query/acc.cgi?acc=GSE3793 (Sebbane et al. 2006), and http://www.ncbi.nlm.nih.gov/geo/query/acc.cgi?acc=GSE16493 (Vadyvaloo et al. 2010). See Materials and Methods section for details.

**Table S3.** Genes of *Burkholderia pseudomallei* K96243 identified by bioinformatic analysis to be expressed at high, medium, or moderately low levels with minimal variation during culture or macrophage infection. The analysis was based on affymetrix data sets http://www.ncbi.nlm.nih.gov/geo/query/acc.cgi?acc=GPL4078,http://www.ncbi.nlm.nih.gov/geo/query/acc.cgi?acc=GSE5495 (Rodrigues et al. 2006), http://www.ebi.ac.uk/arrayexpress/arrays/A-MEXP-206/, and http://www.ebi.ac.uk/arrayexpress/experiments/E-MEXP-334/files/. See Materials and Methods section for details.
